# The Effect of Feeding on *Briareum violacea* Growth, Survival and Larval Development under Temperature and Salinity Stress

**DOI:** 10.3390/biology11030410

**Published:** 2022-03-07

**Authors:** De-Sing Ding, Sheng-Hao Wang, Wei-Ting Sun, Huang-Lin Liu, Chih-Hung Pan

**Affiliations:** Department and Graduate Institute of Aquaculture, National Kaohsiung University of Science and Technology, Kaohsiung 811, Taiwan; dancenet28@gmail.com (S.-H.W.); 1051537103@nkust.edu.tw (W.-T.S.); f110177103@nkust.edu.tw (H.-L.L.); chpan@nkust.edu.tw (C.-H.P.)

**Keywords:** temperature, salinity, artificial polyunsaturated fatty acids, digestive enzymes, settlement, *Briareum violacea*

## Abstract

**Simple Summary:**

Coral reefs are rich in biodiversity. In recent years, the greenhouse effect and heavy rain have threatened the growth and survival of corals. Previous research mainly discussed the harm caused by changes in temperature and salinity to corals. No research has explored effective methods to prevent coral bleaching and death. This study initially found that feeding can promote corals to survive stress response, prevent bleaching or death caused by temperature or salinity changes and significantly improve the number of eggs laid, larval settlement and juvenile development. At present, this technology has been applied to the large-scale production of *Briareum violacea* in coral farms to prevent coral deaths caused by changes in temperature and salinity. In addition, this is very important for the sustainable development of coral reefs.

**Abstract:**

In recent years, climate change has often caused fluctuations in seawater salinity and temperature, which threaten the survival and growth of corals. Effectively improving the stress response to temperature and salinity changes in corals to prevent bleaching is one of the important issues. This study initially explored the use of artificial polyunsaturated fatty acids to assess the ability of *Briareum violacea* to slow bleaching, enhance growth, stabilize larval development and reduce antistress factors (superoxide dismutase and catalase) when they were exposed to temperature and salinity stress. The salinities used in the experiment were 25, 30, 35 and 40 psu, and the temperatures were 20, 25 and 30 °C. It was divided into two parts: Experiment 1—Effects of temperature and salinity and feeding on digestive enzymes, reproduction and stress response of *B. violacea*; Experiment 2—Effects of temperature and salinity and feeding on the settlement and survival of larvae. The results showed that the feeding treatment group reduced the superoxide dismutase, catalase and mortality of corals under stress and significantly improved larval development and larval settlement.

## 1. Introduction

Coral reefs are one of the most important marine ecosystems in the world, with high biodiversity and productivity [1]. Over the past few decades, coral reefs have been endangered by unprecedented environmental changes and man-made pollution [1,2,3]. Corals are considered to be a type of stenohaline osmoconformer and therefore have low tolerance to salinity [4,5,6]. Previous studies have pointed out that corals around the world are facing severe stress responses due to changes in environmental temperature and salinity, which will pose a great threat to the ecological sustainability of coral reefs [7]. Corals of different species may have different tolerances to stressful salinity, which are usually related to the conditions in the coral’s living environment [8,9]. In addition to providing basic nutrition through photosynthetic efficiency, corals are also heterotrophs which can supplement nutrition through feeding. Previous studies have found that the main physiological factors affecting coral growth are photosynthesis, heterotrophic feeding and calcification. In addition, these also include temperature, salinity, pH, competition and predation, larval settlement, etc. All of these factors will cause a stress response in corals and promote coral bleaching or death [1,10,11,12,13,14,15]. According to a study by Harriott (2002), temperature and salinity can affect the physiochemical changes of corals, which can cause stress responses in corals and have a great impact on global coral reef accretion and coral species diversity [16]. Previous research has found that coral in early and adult life stages demonstrate a greater impact of the temperature and salinity stress response [17].

Previous studies have shown that low salinity and high temperatures affect the survival and growth of corals [18], causing a stress response resulting in a decline in photosynthetic efficiency and an inability to provide basic nutrients through zooxanthellae, leading to death [19]. Previous studies have focused on the stress response or physiological changes of coral induced by temperature and salinity changes [19]. However, there is no research on how to improve the stress response of corals to temperature and salinity changes. It is known that the greenhouse effect and heavy rain have caused the seawater temperature to rise and salinity to change, which has caused great harm to the survival of corals.

Effective solutions will have important implications for the ecologically sustainable development of corals and large-scale coral aquaculture.

The area with the richest marine biodiversity of coral reefs is also an important source for the fishery economy [20,21]. Many natural pharmaceutical ingredients found in coral can be used to treat cancer, bacteria, viruses and other diseases [22,23,24]. *Briareum violacea* was found to contain briarane diterpenoids that can fight diseases and are of great importance for marine biodiversity and pharmaceutical applications. In addition, *B. violacea* are of great ornamental value. Therefore, *B. violacea* was used as the main subject of this study. This study is a preliminary study to explore whether *B. violacea* can alleviate the stress response caused by temperature and salinity changes through feeding. In recent years, it has been affected by the greenhouse effect, resulting in salinity changes, environmental pollution, overfishing, coastal development, invasive species, destructive fishing practices, tsunamis overloading tourism, climate change and ocean acidification [7,20,21,25,26,27]. Instantaneous changes in temperature and salinity pose an immediate threat to coral survival in a short time [1,27,28,29]. Changes in temperature and salinity caused 50% of global coral bleaching deaths in 2010 [30,31,32]. According to Burke, Reytar et al., 2011, approximately 75% of the world’s coral reefs are under imminent regional and global threats [20]. Therefore, the sustainability and biodiversity of coral reef ecosystems will be one of the important issues to be addressed as climate change proceeds.

Oxygen is an element required for biological metabolism, which promotes the production of free radicals and reactive oxygen species (ROS). Therefore, when corals are stressed, ROS will increase, causing coral bleaching or death [33,34]. Temperature and salinity changes can increase reactive oxygen species (ROS) in corals, leading to oxidative damage to the coral–zooxanthellae symbiotic system [35,36,37,38]. When environmental changes cause coral stress, antioxidant enzymes are produced to suppress reactive oxygen species. In addition, the formation of oxygen-free radicals can cause cellular defense mechanisms to respond [39]. For example, enzymes such as superoxide dismutase (SOD) and catalase (CAT) act in concert to inactivate superoxide radicals (•O2^−^) and hydrogen peroxide (H_2_O_2_). Therefore, this study will use SOD and CAT as coral antioxidant indicators.

After coral bleaching, zooxanthellae will leave corals and affect the nutrient supply of corals. Through food intake, corals can replenish deficient nutrients. It was expected that under the condition of no zooxanthellae, nutrient supplementation would lead to a resistance to the stress response and prevent the death of corals in a short time. According to a previous study, there are digestive enzymes in *Goniopora columna*, among which protease content is the highest, suggesting that providing suitable feed can improve the survival and growth of coral [40]. It is known that feed supply can be applied to nutritional supplements of coral. If the method of improving the response to temperature and salt stress can be applied, it will be of great help to the ecological sustainability of coral reefs. In addition, the greenhouse effect also threatens coral reproduction [41,42]. Previous studies have shown that temperature, salinity and dissolved oxygen can stimulate coral reproduction [43]. Therefore, Experiment 1 in this study explored whether feeding *B. violacea* could promote their access to basic nutrients to resist the stress response caused by temperature and salinity changes, slow down the mortality rate and evaluate whether the spawning rate could be improved after feeding. In addition, changes in temperature and salinity are also important influencing factors during larval settlement and juvenile development of *B. violacea*. Therefore, Experiment 2 was conducted to explore whether feeding can improve larval settlement and juvenile development of *B. violacea* in the context of temperature and salinity stress responses.

## 2. Materials and Methods

### 2.1. Source of Coral Samples

The 200 *B. violacea* colonies used in this experiment were obtained from the Taiwan Coral King coral farm (Kaohsiung, Taiwan), a legal coral farm (CITES No. FTS507 W0153796). *B. violacea* is a kind of octocoral, an Alcyonacea; the body tissue structure is composed of calcareous spicules, endoderm, mesohyl and ectoderm. Each polyp has a mouthpart in the middle which is concave to form a stomodaeum and a pharynx. Each polyp is linked by coenosarc and can grow flat on the reef surface. Coral farms raise corals with a salinity of 30 ± 0.5 psu and a temperature of 25 ± 0.5 °C. Corals were housed in a recirculating filtered seawater system fish tank (60 × 35 × 30 cm) and fed. Purple lights of 400–430 nm (HME Block 2 Series) were installed 30 cm above the water surface as the main light source. Water pumps were also installed to create a flow of water. After 14 days of acclimation and tissue repair, the healthy corals were segmented into 5-polyp colonies and fixed on porous foundation stones with coral glue. There were 10 colonies per treatment group. All experiments were repeated three times. Therefore, each group involved a total of 30 colonies (*n* = 30 colonies). After waiting 72 h, the coral polyps were fully stretched and the experiment was begun. Water quality was monitored and controlled to be within a safe range every day, as shown in Table 1.

### 2.2. Experimental Conditions

This experiment was modified by referring to the method of [44]. Salt measurements were performed using a salinometer (ATAGO Handheld Refractometer). Natural seawater at 35 psu was used for the control group. RO pure water was used to reduce the salinity of seawater to 30 and 25 psu, and coral sea salt was used to increase the salinity to 40 psu (40, 35, 30 and 25 psu) before being placed into an aquarium tank (60 × 35 × 30 cm). The temperature was controlled at 20 ± 0.5 °C, 25 ± 0.5 °C and 30 ± 0.5 °C. The temperature was controlled by using a Johnlen 300 W water heating and chilling machine (T and F 1/3 HP) to keep the temperature constant. Water quality was measured once a day, and temperature and salinity were constantly controlled diurnally to ensure experimental stability.

### 2.3. Feed Source

The feed diet contained a mixture of intact and hydrolyzed marine and terrestrial ingredients (commercial-in-confidence formulation, details not provided); feed formula published by Ding [40]. The feed formula included artificial polyunsaturated fatty acids (PUFAs) rich in animal protein (i.e., a formulated diet combining animal protein and sodium alginate with probiotics). Please refer to Table 2 for the nutritional composition of the feed. During the experiment, corals were fed 5% of their body weight once a day. The feeding treatment group was represented by Y and the nonfeeding group was represented by N. This study evaluated the coral feed’s effects on nutrient uptake with reference to changes in the body composition content of coral levels by feeding in accordance with the experimental method of [45]. Changes in body composition between the feed and no-feed groups were used to assess whether corals ingested feeds. Protein, lipid and glucose assays were performed 1 h after feeding. Half of the seawater was replaced before feeding to remove the remains of feed suspension, and seawater adjusted for temperature and salinity was used to avoid changes in temperature and salinity. During the experiment, the daily feeding time was fixed at 08:00 in the morning.

### 2.4. Experiment 1: Effects of Temperature, Salinity and Diet on Body Composition, Digestive Enzymes, Reproduction and Stress Response of Briareum violacea

#### 2.4.1. Determination of *Briareum violacea* Growth and Survival

*Briareum violacea* growth was determined by total weight and polyp count as described by [46,47]. The tissue’s dry weight was measured. Algae were brushed, and the surface of the coral was dusted off. Coral was placed in plastic Petri dished and weighed using an electronic balance. The larger polyps of *B. violacea* were used for macroscopic examination. Calculations were made once a week, and photographs were taken to record the increased number of new polyps using a Canon EOS 750D camera (Tokyo, Japan). The experiment was carried out for 8 weeks. After the experiment, the specific growth rate (SGR), mean and standard deviation were calculated, and the body composition, digestive enzymes, SOD and CAT were detected. In addition, some corals’ egg production and egg size were observed for 365 days to evaluate the effects of feeding and temperature and salinity on coral reproduction. The amounts of tissue and skeleton (dry weight) and polyps were measured every 7 days during the 8-week experiment. Specific growth rate (SGR), mean value and standard deviation were calculated after the experiment. The SGR of the coral was measured using the following formula:

(1) $$\mathrm{SGR}\;(\%\;{\mathrm{Day}}^{-1})=\left(\frac{wf-wi}{\Delta t}\right)\;\times \;100$$

where *wf* is the final weight of the coral (g), *wi* is the initial weight of the coral (g) and Δt is the experimental time in days.

After the experiment, to calculate the survival of corals, the disappearance of all polyps was used as the basis for death. The mean and standard deviation (SD) of the survival rate were calculated. The survival rate of the coral was calculated using the following formula:

Survival rate (%) = (final number alive/number of initial samples) × 100

#### 2.4.2. Analysis of Coral Body Composition

Through this experiment, the effects of changes in temperature and salinity on the digestive enzymes and body composition of *B. violacea* were analyzed. A scalpel was used to scrape coral tissue off foundation stones. *B. violacea* were sonicated and investigated, and the protein concentration was measured by a Bradford protein assay kit (Ameresco, Fountain Parkway, Solon, OH, USA) with bovine serum albumin as a protein standard. Fat content analysis was performed according to official methods [44,48], and lipids were extracted from *B. violacea* by hexane; then, subsamples were transferred to test tubes and evaporated to dryness. The obtained total lipid weights were converted to micrograms. The calculation formula is expressed as follows:

(2) $$\mathrm{Lipid}=\frac{Wi-Wo}{S}\times 100$$

where *Wo* = constant weight of the aluminum cup (g), *Wi* = extracted oil contained in the aluminum cup weight (g) and S = sample weight (g). Carbohydrates were measured based on [49,50], with glucose serving as the reference material. An absorption value of 505–660 nm was used to determine the glucose content. The formula for glucose content derivation is expressed as follows:

glucose (µg/mL) = (A (sample with colorimetric test − sample)/A (standard tube)) × Glucose standard concentration (µg/mL)

After the experiment, the mean and standard deviations were calculated, and statistical analysis was performed. 

#### 2.4.3. Analysis of Digestive Enzymes

Enzyme extraction was performed using the method of Sun et al. (2007) [51]. Protease and lipase extractions were performed using 10 mM sodium citrate buffer (pH 7.0) in a cold environment. Each coral was first rinsed in buffer solution and then added to 10 times the volume of buffer solution, placed on ice for grinding and subsequently centrifuged (10,000× *g*) at 4 °C for 10 min. Thereafter, the supernatant was collected and stored at −20 °C. The protease content was analyzed using the method of Sun et al., (2007) [51], which involved adding 1 mL of casein to 0.5 mL of enzyme extract, incubating the mixture for 15 min and then adding 1.5 mL of 10% trichloroacetic acid. After centrifugation (6000× *g*) at 4 °C for 10 min, the supernatant was collected and 5 mL of 0.55 M Na_2_CO_3_ and 1 mL of Folin phenol-staining reagent were added. The absorbance value at 680 nm was observed.

Lipase content was analyzed using the method of Borlongan (1990) [52]. To 1.5 mL of olive oil, 1.5 mL of Tris–HCl (0.1 M buffer, pH 8.0) and 1 mL of enzyme extract were added, and the solution was then shaken at 37 °C for 6 h. Thereafter, 95% alcohol was added to terminate the reaction, and thymolphthalein containing 0.9% alcohol was used as the indicator. The mixture was then titrated with 0.01 N NaOH until the solution turned brown.

Amylase content was analyzed using the method of Bernfeld (1955) [53]. To 0.05 M phosphate buffer solution (pH 7.0), 1 mL of 2% (*w*/*v*) starch solution was added, and the mixture was maintained at 25 °C for 5 min. Then, the enzyme extraction was added, and the mixture was left to react at 20–60 °C. Subsequently, 2 mL of dinitrosalicylic acid reagent was added before the reaction was stopped in a boiling water bath for 5 min, and the mixture was then cooled. The absorbance at 520 nm was measured as per the maltose standard. Amylase activity was determined as maltose content per milligram of protein per minute. At the end of the experiment, means and SDs were calculated.

#### 2.4.4. Analysis of Zooxanthellar Density and Chlorophyll *a*

At the end of the 8-week experiment, the coral tissues were homogenized and the number of zooxanthellae in *B. violacea* was observed and calculated with a blood cell counter according to [54]. This study refers to LaJeunesse et al. (2018), and in the following work’s description, Symbiodiniaceae is represented by the classical term zooxanthellae [55]. The zooxanthellae density is expressed as number per polyp. For the determination of the chlorophyll *a* content, according to the methods of [56], fresh coral tissue (0.5 g) was first homogenized, and then 10 mL of 90% acetone was added to extract chlorophyll *a* before being left to stand for 24 h at 4 °C under all-black conditions. The absorption spectra were measured at 630 and 664 nm using a Hitachi U-2000 spectrophotometer (Tokyo, Japan), and the concentration was calculated using the equations of [57]. The chlorophyll *a* content was measured in the immediately sampled *B. violacea* colonies as micrograms of chlorophyll *a* per gram of the wet weight of colony tissues.

#### 2.4.5. Antioxidant Enzyme Analysis

##### Preparation of Coral Tissue Solution

The detection method of [58] was used. Coral tissue was homogenized and placed in nine volumes of ice-cold extraction buffer (20 mM phosphate buffer, 1 mM EDTA, 0.1% (*v*/*v*) Triton X-100; pH 7.4). Crude extract was sonicated (3 s each, five times) and centrifuged (4 °C, 5 min and 12,000× *g*) and the resulting tissue solution was used for protein and enzyme assays.

##### Superoxide Dismutase Detection

SOD activity was assayed spectrophotometrically as described by Cheng et al. (2021), [38,59]. Bovine erythrocytic was used as the activity standard for SOD and CAT for each treatment group (Sigma, NY, USA). Next, 15,000 µL of the tissue solution was placed into the test tube, and then 10,000 µL of 0.1 mole PBS buffer was added and centrifuged at 1500× *g*, 30 min. After centrifugation, the supernatant solution was taken as the test sample. The enzyme activity was expressed as units (U) per milligram of protein, and the assay was performed at a constant temperature of 25 °C. The Bradford assay [60] was used for protein detection.

##### Catalase Detection

CAT activity was detected by Sigma reagent, and the experimental operation used Main, Ross and Bielmyer’s (2010) [61] catalase measurement. First, the sample preparation was conducted. Then, 15,000 µL of the tissue solution was placed into the test tube, and then 10,000 µL of 0.1 mole PBS buffer was added and centrifuged at 1500× *g*, 30 min. After centrifugation, the supernatant solution was taken as the test sample. The sample was thoroughly mixed with 25 µL of a blank colorimetric assay substrate solution, and the solution was left to stand for 1~5 min. Afterward, 5 µL of the sample was placed into a test tube, 70 µL of 1 × assay buffer was added, and then 75 µL of blank 1× assay buffer was added. Next, the sample was thoroughly mixed with the blank, and 25 µL of colorimetric assay substrate solution was added before being left to stand for 1~5 min. Next, 900 µL of stop solution was added to the sample and blank before being mixed upside down. Then, 10 µL of reaction mixture was moved into another test tube, after which 1 mL of color reagent was added, which was left for 15 min at room temperature before the absorbance value at OD 520 nm was measured.

Calculation formula:

ΔA = A_sample_ − A_blank_ (3)

CAT (µmoles/min/mL) = (ΔA × d × 100)/(V × t)(4)

where d = diluted multiples, t = the reaction time and V = the volume of the sample.

##### Protein Concentrations

*Briareum violacea* tissue was first sonicated, and protein quality was measured using the Bradford protein assay kit from Amresco, Solon, OH, USA, using bovine serum albumin as a standard.

#### 2.4.6. Egg Production Calculation and Egg Size Measurement

From the onset of the experiment, the corals in the aquarium tank were observed daily for spawning (a total of 365 days of observation). The observed eggs were collected using plankton nets (300 mesh), and the number of eggs was calculated according to the visual calculation method [62]. A microscope was used to measure egg size. Egg volume was calculated using the elliptical integral equation:

V = (4/3)πab^2^ (5)

where a = ½ egg length and b = ½ egg width [62,63].

### 2.5. Experiment 2: Effect of Temperature, Salinity and Diet on the Settlement and Survival of Briareum violacea Larvae

#### 2.5.1. Experimental Conditions

The temperature and salinity were controlled at 25 psu, 30 psu, 35 psu and 40 psu. At 20 ± 0.5 °C, 25 ± 0.5 °C and 30 ± 0.5 °C, a glass tank (60 × 35 × 30 cm) was used. One group was fed (Y) and the other group was not fed (N). Each treatment group had 100 eggs and was fed 3% of the egg weight. Each group was a triplicate. After spawning, coral eggs were collected and put into the aquarium tank (*n* = 300 eggs). On Days 8, 10, 12, 14, 16 and 18 of the experiment, the settlement and development of coral were checked and the number of larvae was calculated. The determination method of settlement was that the attached larvae formed round, flat tissues or single polyps attached to the aquarium tank wall. This experiment referred to [64] as the larval settlement method for improvement. A TG4 camera (Olympus, Beijing, China) was used to record the developmental patterns of the juvenile period. The experiment lasted for 80 days, until it was observed that the coral larvae polyps began to divide and proliferate.

#### 2.5.2. Determination of Zooxanthellae Number and Chlorophyll *a* in *Briareum violacea* Larvae

*Briareum violacea* polyps cultured for 80 days were scraped off of the aquarium tank with a scalpel and detected by referring to the detection method of Section 2.4.4, symbiotic algae number and chlorophyll *a*.

### 2.6. Statistical Analysis

Data were obtained from two independent experiments, and the final results are presented as the mean ± standard deviation (SD). One-way analysis of variance and Duncan’s multiple range test were used to determine the statistical significance for fed (Y), not fed (N), SOD, CAT, survival of *B. violacea*, zooxanthellar density and chlorophyll *a* and larval settlement content in *B. violacea*. A *p* value of < 0.05 was considered significant. All statistical analyses were performed using IBM SPSS Statistics 20.

## 3. Results

### 3.1. Experiment 1: Evaluation of the Response of Feeding Bait to Thermohaline Stress in B. violacea

#### 3.1.1. Effects of Temperature, Salinity and Feeding on *B. violacea* Growth and Survival

Body composition analysis was performed 1 h after feeding to evaluate the changes in coral body composition, proteins, lipids and glucose with and without feeding (Table 2). The results showed that the protein content of feeding for 1 h was 1.32 times higher than that of no feeding, while lipids and glucose increased by 1.18 and 1.54 times, respectively. According to the preliminary results of the experiment, it was found that feeding can indeed increase protein, lipid and glucose content in corals.

**Figure 1:**
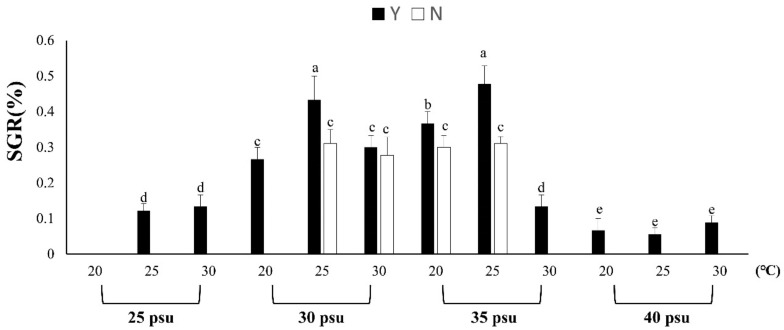
The effect of feeding on *Briareum violacea* SGR during temperature and salinity stress. Different Latin alphabet letters indicate significant differences among groups (*p* < 0.05). The values are expressed as the means ± SDs (*n* = 30 colonies). Y: feeding treatment group, N: nonfeeding treatment group.

The growth experiment of coral at different temperatures and salinities showed that the growth of coral at salinities of 30 psu and 35 psu and SGR was higher at 25 °C (Figure 1). The above temperature and salinity ranges are suitable for coral growth. However, the growth of Y was significantly higher than that of N, indicating increased growth through feeding. However, according to the results, no growth was found in each treatment group with a low salinity of 25 psu-N. In addition, the Y growth rate did not increase at 20 °C, 25 °C or 30 °C compared with N. In the high-salinity, 40-psu condition, it was found that the growth rate of the N treatment group was 0, and the SGR of each Y treatment group increased by 0.07, 0.06 and 0.09 times compared with the N group. At salinities of 30 and 35 psu, the polyps’ growth rate was observed to be faster at a temperature of 25 °C, but there was no increase in the polyps of each group at −20 °C (Table 3). Therefore, the results show that corals in low salt, high salt, low temperature and high temperature environments can cause coral stress responses, resulting in impacts on coral growth. Table 4 shows that the survival rate of the treatment group with feeding (Y) was 100%, while the unfed treatment group (N) had a 100% survival of 30 psu, 25 °C; 30 psu, 30 °C and 35 psu. The survival rate was 100% at 25 °C. In conclusion, feeding can effectively prevent coral growth retardation caused by temperature and salinity stress responses. It can also prevent coral from dying due to the stress response.

#### 3.1.2. Evaluation of Resistance to Stress by Feeding

**Figure 2:**
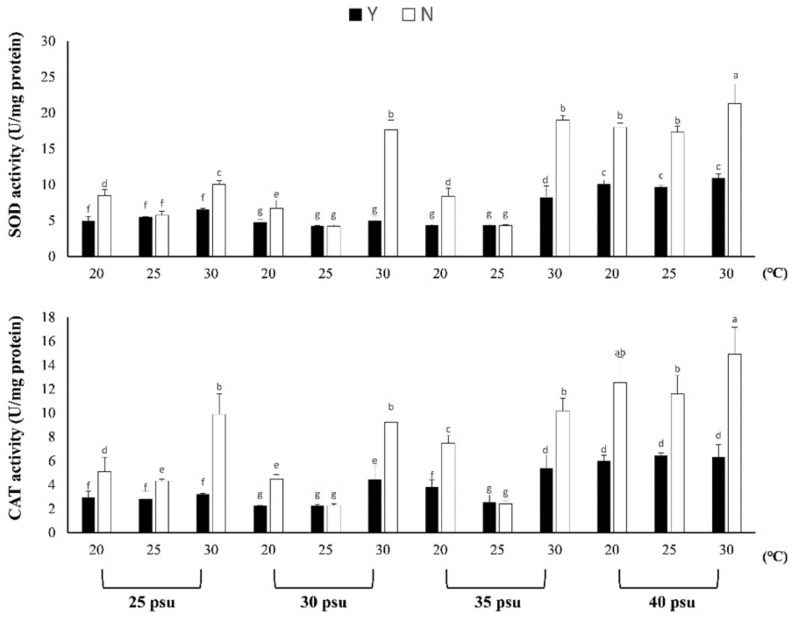
The effect of feeding on *Briareum violacea* SOD and CAT under temperature and salinity stress. Different Latin alphabet letters indicate significant differences among groups (*p* < 0.05). The values are expressed as the means ± SDs (*n* = 30 colonies). Y: feeding treatment group, N: nonfeeding treatment group.

In Figure 2, the results showed that the SOD value in each treatment group with feeding (Y) was significantly lower than that without feeding (N) under temperature and salinity stress responses (F23,48 = 40.849, *p* < 0.005), indicating that feeding can supplement basic energy to resist stress responses caused by the environment. At 30 psu, the SOD difference between Y and N at 30 °C was 3.56 times. At 35 psu and 30 °C, the SOD of Y and N differed by 2.31 times, and the SOD of each temperature treatment group at 40 psu differed by 1.78, 1.80 and 0.51 times, respectively.

The results of the CAT showed that the salinity was 30 and 35 psu; there was a lower CAT at a temperature of 25 °C and there was no significant difference between the Y and N treatment groups (Figure 2). In the other treatment groups, there was a 1.53–2.36-fold decrease in Y compared with N. According to the results, in the temperature and salt stress response, although the oxidation enzyme in the Y treatment group also increased, it was significantly lower than that in the N treatment group.

The results of different salinities on zooxanthellae and chlorophyll *a* are shown in Table 4. Under the thermohaline stress response, zooxanthellae and chlorophyll *a* had a downward trend, indicating that thermohaline stress promotes zooxanthellae to leave the coral endoderm, resulting in coral bleaching.

Although the results show that the number of zooxanthellae in each treatment Group Y may be higher than that in treatment Group N, there was still a significant decrease compared with the 30 to 35 psu and 25 °C treatment groups. In conclusion, temperature and salinity can cause a stress response and bleaching of coral. Through feeding nutritional supplements, basic nutrition of coral can be improved, and the ability to resist the stress response can be improved to avoid bleaching or death of coral caused by environmental changes.

#### 3.1.3. Effects of Temperature and Salinity Stress on Body Composition and Digestive Enzymes

**Figure 3:**
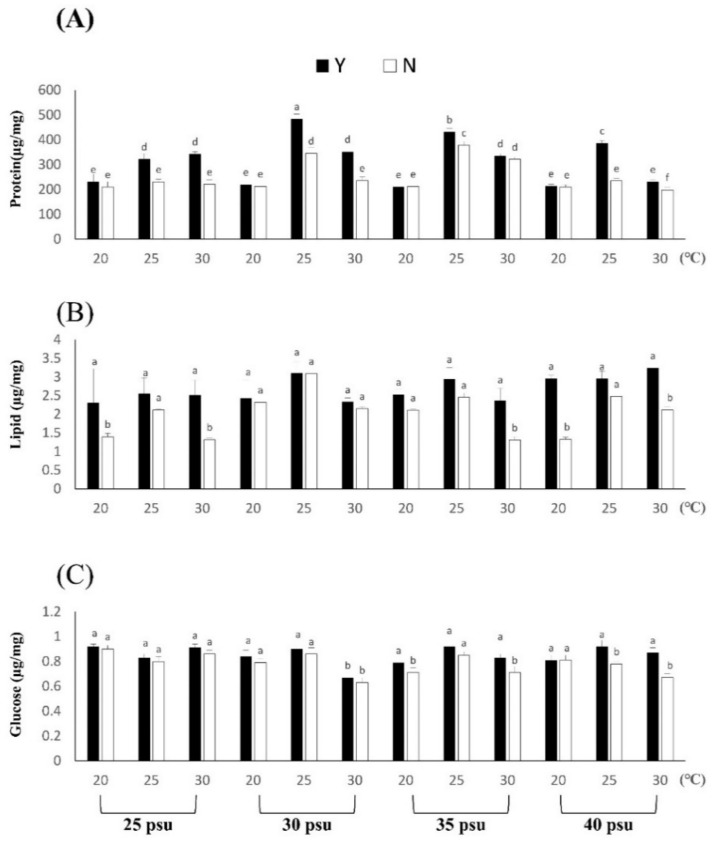
The effect of feeding on *Briareum violacea* body composition during temperature and salinity stress. Different Latin alphabet letters indicate significant differences among groups (*p* < 0.05). The values are expressed as the means ± SDs (*n* = 30 colonies). (**A**): protein, (**B**): lipid, (**C**): glucose.

**Figure 4:**
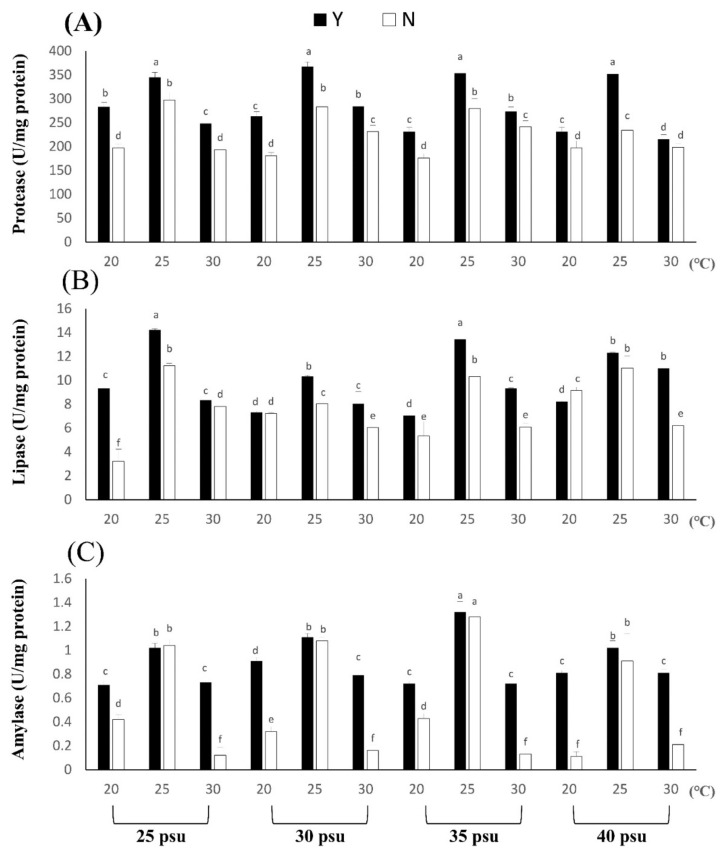
The effect of feeding on *Briareum violacea* digestive enzymes during temperature and salinity stress. Different Latin alphabet letters indicate significant differences among groups (*p* < 0.05). The values are expressed as the means ± SDs (*n* = 30 colonies). (**A**): Protease, (**B**): lipase, (**C**): amylase.

The experimental results showed that different temperatures and salinities affected the changes in *B. violacea* body composition and digestive enzymes (Figure 3 and Figure 4). According to the experimental results, the protein content of the coral body composition is the highest, with 30 psu at 25 °C and 484.21 ± 19.35 μg/mg. There was no significant difference in protein content under different salinity at a low temperature of 20 °C, which may be caused by the slow uptake and metabolism of coral nutrients at low temperatures. The protein content in each N treatment was significantly lower than that in Y. The difference between Y and N was 0.99 to 1.55 times, and the biggest difference was found in the 40 psu group at 25 °C (*p* < 0.05). High salinity and a low-temperature environment may affect the basic nutrition provided by zooxanthellae to corals, but corals can directly absorb and utilize bait, feeding to overcome the damage caused by the stress response. The lipid and glucose contents are shown in the table, and the difference between Y and N was 1.00 to 2.23 times and 1.02 to 1.18 times, respectively. The largest difference in lipid content was 40 psu at 20 °C (*p* < 0.05), and the largest difference in glucose was 40 psu at 25 °C (*p* < 0.05). In summary, the body composition content of each treatment Group Y was higher than that of N (*p* < 0.05). Feeding can increase the protein, lipid and glucose contents of coral under temperature and salinity stress response environments. After the experiment, the coral digestive enzymes were tested, and it was found that the corals had high protease content, while the lipase and amylase contents were lower. Therefore, the main nutrient that *B. violacea* take in should be protein. The results of Figure 4 show that the Y salinity treatment groups had higher protease activities at 25 °C, which were 345.21 ± 10.10, 367.31 ± 10.03, 353.30 ± 7.03 and 352.14 ± 10.32 U/mg protein, respectively. In each salinity treatment group, Y and N differed by 1.16, 1.30, 1.26 and 1.50 times, respectively, at 25 °C. Although the content of lipase in corals was relatively low, except for the 30 psu, 20 °C groups, there was no significant difference, and other Y groups still had significantly higher lipase contents than N treatment groups.

In the amylase test, it was found that the content in *B. violacea* was very small, ranging from 0.12 to 1.32 U/mg protein. Although the content of amylase was low, the temperature and salinity stress reaction still affected the activity of amylase. The largest difference was for 40 psu at 20 °C; the difference between Y and N was 7.36 times.

#### 3.1.4. Spawning Volume and Egg Size

**Figure 5:**
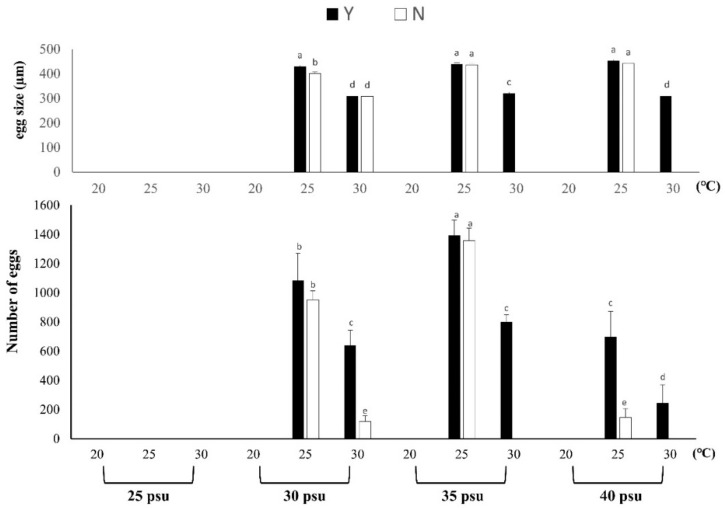
The effect of feeding on *Briareum violacea* egg size and number of eggs under temperature and salinity stress. Different Latin alphabet letters indicate significant differences among groups (*p* < 0.05). The values are expressed as the means ± SDs (*n* = 30 colonies). Y: feeding treatment group, N: nonfeeding treatment group.

The annual spawning quantity of *B. violacea* under different temperature, salinity and feeding conditions showed that the spawning quantity was the highest at 35 psu at 25 °C; Y and N were 1393.00 ± 106.04 in number and 1357.07 ± 87.04 in number, respectively, and there was no significant difference between the two treatment groups (Figure 5). At 30 psu, the 30 °C spawning quantity for Y was 5.27 times greater than for N at 35 psu, and the 30 °C spawning quantity for Y was 800; N was 0, and the result shows that the spawning quantity of the Y treatment group in a high-temperature environment was significantly higher than that of the N treatment group. The spawning amount at 40 psu and 25 °C for Y was 4.78 times greater than for N. No spawning was observed under the three temperature conditions of low salinity at 25 psu, and no spawning was observed at a temperature 20 °C for each salinity treatment group. The average egg size was 385.1 ± 64.37μm. According to Figure 5, the egg size of each treatment group was 30 psu, 35 psu and 40 psu; the egg size was the largest at 25 °C, which was significantly different from that of the other groups (*p* < 0.05). Among them, the egg diameter of Y at 35 psu and 25 °C was 1.07 times larger than that of N. According to the results, the diameter of eggs produced by Y at a high temperature of 30 °C was smaller, with an average of 310 to 320 μm. Therefore, under feeding conditions, temperature and salinity stress may affect the development and size of *B. violacea* eggs.

### 3.2. Experiment 2: Larval Settlement and Larval Development of B. violacea

#### 3.2.1. Larval Settlement

**Figure 6:**
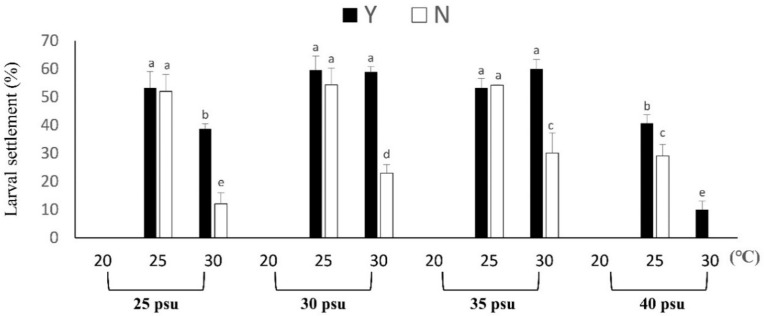
The effect of feeding on *Briareum violacea* larval settlement during temperature and salinity stress. Different Latin alphabet letters indicate significant differences among groups (*p* < 0.05). The values are expressed as the means ± SDs (*n* = 300 eggs). Y: feeding treatment group, N: nonfeeding treatment group.

The results of *B. violacea* larval settlement are shown in Figure 6. The results are shown at 25, 30 and 35 psu; the larval settlement at 25 °C was the best. The comparison of the Y and N treatment groups with salinities of 25, 30, 35 and 40 psu at a temperature of 30 °C showed that the differences were 3.20 ± 0.50, 4.00 ± 1.02, 2.00 ± 1.00 and 10.04 ± 3.10 times, respectively. Therefore, the larval settlement of each treatment Group Y was better than that of N at 30 °C. The larval settlement of the N group is 0%, and that of the Y group was 10.04% when the salt content was 40 psu at 30 °C. However, no larval settlement was found at a low temperature of 20 °C. After 6 months of cultivation, the survival rate of coral is shown in Table 5. The Y treatment group at 35 psu and 25 °C had a survival rate of 45.00 ± 5.00%, which was significantly different from that of the other groups (*p* < 0.05). Compared with the N group, the survival rate increased by 1.17 times.

#### 3.2.2. Larval Development of *B. violacea*

**Figure 7:**
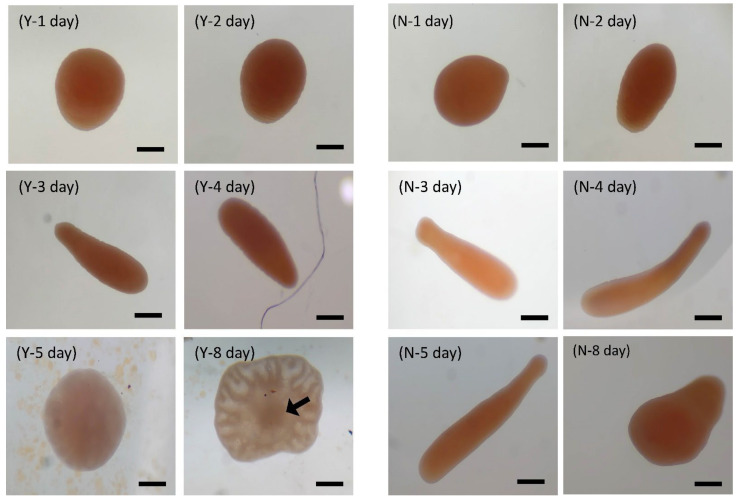
The effect of feeding on *Briareum violacea* larval development during temperature and salinity stress (scale bar: 100 μm). 1 day: the eggs are round and sticky and adhere to the surface of the aquarium tank. Two days: the planula larva, which is in the shape of a drop, begins to crawl slowly. Three days: the body of the planula larva is elongated; the tip is the head and it crawls and moves quickly. Four days: in the Y group, the planula larva has a body length of about 730 μm and a relatively slender body. In the N group, the planula larva has a body length of about 500 μm and a round head. Five days: in the Y group, the planula larva has begun to prepare settlement. In the N group, the planula larva slows down and its body length is about 800 μm. Eight days: in the Y group, the settlement is complete, and the larva is attached to the aquarium tank. The arrow points to the undifferentiated polyps. In the N group, there is slower development and no settlement yet. Y: feeding treatment group, N: nonfeeding group.

**Figure 8:**
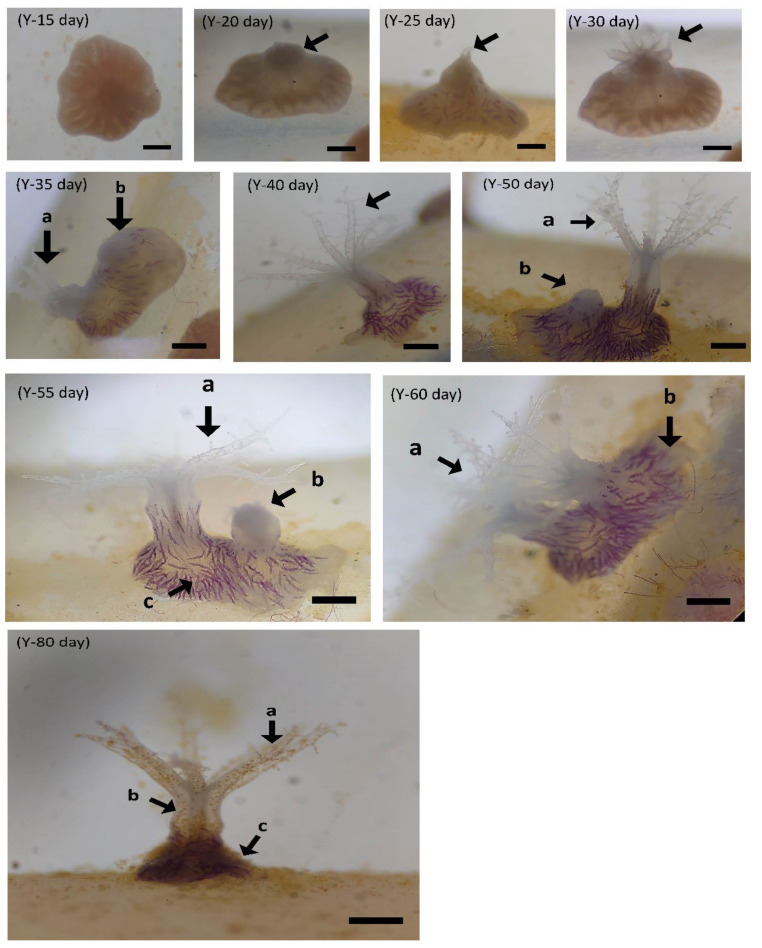
The effect of feeding on *Briareum violacea* juvenile development during temperature and salinity stress. Y, feeding treatment group (scale bar: 100 μm). Fifteen days: in the Y group, the polyps’ organization begins to expand. Twenty days: in the Y group, the middle is raised; the arrow points to the undifferentiated polyps. Twenty-five days: in the Y group, a tentacle appears in the middle bulge. Thirty days: in the Y group, it can be observed that the development of the polyp is completed (the arrow points to it). Thirty-five days: in the Y group, coenosarc begins to expand and grow outward (indicated by the arrows; a, original polyp; b, coenosarc tissue). Forty days: in the Y group, polyps are about 300 μm long and spicules can already be observed in polyps. Fifty days: in the Y group, polyps begin schizogamy (indicated by the arrows; a, original polyp; b, new schizogamy polyp). Fifty-five days: in the Y group, raised tissue forms next to the polyps (indicated by the arrows; a, original polyp; b, new schizogamy polyp; c, coenosarc tissue). Sixty days: in the Y group, polyps split into two polyps (indicated by the arrows; a, original polyp; b, new schizogamy polyp). Eighty days: in the Y group, there are many golden yellow zooxanthellae in the polyps’ tissue (indicated by the arrows; a, tentacles containing zooxanthellae; b, polyp; c, coenosarc tissue).

**Figure 9:**
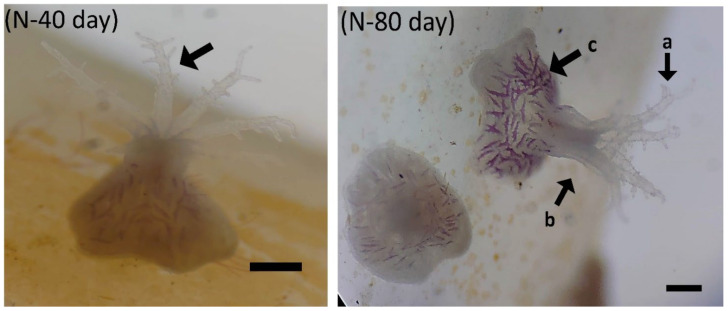
The effect of feeding on *Briareum violacea* juvenile development during temperature and salinity stress. N, nonfeeding group (scale bar: 100 μm). Forty days: in the N group, the polyp is about 200μm long and spicules have not been observed (indicated by the arrow a, tentacles of polyp). Eighty days: in the N group, no zooxanthellae were found in polyps or coenosarc tissues (indicated by the arrows; a, tentacles; b, polyp; c, coenosarc tissue). The polyp has no schizogamy at all.

Coral larval development was observed, as shown in Figure 7. Larvae grew faster under the conditions of Y-group temperature and salinity stress. Eggs can develop into planula larvae 2 days after hatching. On the third day, the body was long and crawling, and on the eighth day, larval settlement began. On the tenth day, the body was round and flat, and polyps were observed after 15 days. The tentacles were not fully developed until 35 days after hatching. In the N treatment group, it was found that the eggs successfully developed into planula larvae but were hindered in the development stage. The shape was similar to that of water droplets or short rods, as shown in Figure 7, which was different from the elongated shape of the larvae in the Y treatment group. Death occurred after four days of temperature and salinity stress tests. It was observed that polyps in the Y group (40 psu; 30 °C) developed complete polyps and began to divide and proliferate after 35 days under thermohaline stress (Figure 8). The N treatment group was observed for 80 days and it was found that no zooxanthellae were found in the body, and no polyps split (Figure 9). The results show that feeding can promote the smooth development of planula larva into polyps in high-temperature and high-salinity environments, and can promote polyps to split after 35 days.

#### 3.2.3. Larval Zooxanthellae and Chlorophyll *a*

*B. violacea* larval zooxanthellae test results are shown in Table 5. In each treatment group at 25 °C, the Y treatment group (25, 30, 35 and 40 psu) increased by 1.34, 3.94, 1.85 and 8.45 times, respectively, compared to the N treatment group. The biggest difference was at 35 psu and 30 °C, where Y significantly increased by 11 times greater than N (*p* < 0.05). The Y treatment group (25, 30, 35 and 40 psu) of chlorophyll *a* increased by 1.75, 2.75, 1.83 and 1.6 times, respectively, compared to the N treatment group. According to the results, feeding can significantly improve the *B. violacea* bleaching problem caused by larval resistance to temperature and salinity stress.

## 4. Discussion

This study found that under temperature and salinity stress, feeding can enhance coral growth, survival, egg laying amount and larval development. The stress response caused by the environment will cause the zooxanthellae to excrete the coral endoderm, causing the coral to be in a stress response and to be unable to eat enough nutrients, causing death [18,19]. According to a study [19], corals can cause bleaching outside of suitable temperatures and salinities. This is consistent with our research findings. Therefore, through our preliminary research, we found that feeding during temperature and salinity stress can effectively enhance the survival and growth of *B. violacea*. This has considerable implications for coral restoration and large-scale coral aquaculture. Previous research has focused on the impact of environmental stress on corals but has not yet explored a solution. Our recent research found that, in addition to the basic nutrients provided by zooxanthellae, corals can also supplement their nutrition through food intake and accelerate their growth by up to 1.3 times [40]. According to the results, the survival rate of the Y treatment group was 100%, while the N treatment caused a stress response, resulting in albinism and death. In addition, the antistress factors SOD and CAT in the Y treatment group were lower than those in the N treatment group, indicating that feeding has an inhibitory effect on the temperature and salinity stress response of corals. Previous studies have found that unsuitable temperatures and salinities cause coral zooxanthellae to leave the endoderm and cause coral bleaching. Because coral contains a lot of bacteria and archaea, which increase nitrogen fixation when the temperature rises, this leads to the proliferation of zooxanthellae in the coral and the destruction of nitrogen: phosphorus ratios in the coral, causing coral stress [65]. At this time, SOD and CAT increase significantly [35,44]. The results of this study show that the conditions of group Y can still cause a stress response in corals, triggering zooxanthellae to excrete the endoderm, but that they can effectively stabilize the values of antioxidant enzymes SOD and CAT, which means that proper feeding can enhance the antioxidant capacity of *B. violacea*. However, under low salinity or low temperature conditions without feeding, the growth and reproductive capacity of corals is very poor. This is similar to the findings of [44] that when the temperature is as low as 20 °C, most of the corals will die, and when the temperature is 30 °C, some corals will bleach or die. Therefore, the improvement of the stress response to low temperature or low salinity in corals needs further research in the future.

In terms of spawning volume and number of eggs, the Y treatment group had a significantly higher volume and number of eggs than the N treatment groups. Temperature and salinity stress caused coral spawning to decrease or not occur at all. Studies have found that the annual spawning volume is 0 at a low temperature of 20 °C or a high temperature of 30 °C. A high temperature of 30 °C can promote an increase in egg production through feeding, but a low temperature of 20 °C will still inhibit coral spawning. A *Platygyra acuta* temperature and salinity stress response study found that when the salinity was 26 psu, normal embryonic development was reduced by >80%, and when the salinity was 22 psu, it caused 100% abnormal development [17]. Salinity below 28 psu will result in *Acropora millepora* eggs becoming unfertilized [66]. This may be because when corals are exposed to temperature or salinity stress, the zooxanthellae in the endoderm are excreted, resulting in insufficient coral nutrition, which affects reproduction, or the eggs cannot resist the stress response [17]. In addition, the egg diameter resulting from the Y treatment was also larger than that resulting from the N treatment. It may be that nutritional supplementation affects the development of eggs and improves the quality of eggs.

The larval settlement of the N treatment group was 0% when high salinity was 40 psu at 30 °C, and that of the Y treatment group was 10.04%, which means that larval settlement can be promoted by feeding when the high temperature is 30 °C. Previous studies have found that changes in high temperature and salinity can cause the albinism or death of *Cyphastrea microphthalma* and *Platygyra daedalea* [67]. In an environment with a temperature increase of 3 °C, it will have no effect on the fertilization of *Platygyra acuta* [17]. This may be caused by different coral species’ responses to temperature and salinity stress. Current research contends that the factors that will induce coral settlement are temperature, salinity, pH and dissolved oxygen [68]. This study found that the Y treatment group at 30 °C and at 25 psu to 35 psu can indeed increase larval settlement by 2.59% more effectively than the N treatment group. However, no larval settlement was found at a low temperature of 20 °C, so a low-temperature environment was not suitable for larval settlement in corals. In addition, most corals have no zooxanthellae in their bodies before larval settlement, and they need to ingest to achieve basic nutrients [69,70,71]. The combination of larvae and zooxanthellae may promote the generation of ROS, leading to lipid peroxidation and DNA damage [72,73]. If subjected to temperature and salinity stress, it is easy to cause the death of the larvae. This study found that the survival rate of the Y treatment group was 100%, while the N treatment group’s survival rate was 0%. When the environmental temperature and salinity are appropriate, the survival of *P. damicornis* larvae is attributed to the zooxanthellae in the endoderm, which provide basic nutrients [74]. *Fungia fungites* larvae can tolerate temperatures up to 34 °C, while *Lithophyllon repanda* larvae cannot survive temperatures above 31 °C [75]. Previous research indicated that corals with alleles are more tolerant of temperature and salinity changes. Therefore, coral bleaching may be controlled by multiple quantitative trait loci [65]. However, there are few previous studies on the mitigation of stress response and bleaching in corals. This study initially found that of the effects of unsuitable temperatures and salinities such as bleaching and stress responses can be alleviated through feeding, which can also promote juvenile development. There was feeding to allow *B. violacea* to develop smoothly at 30 °C. Therefore, when corals are exposed to temperature and salinity stress during reproduction, to achieve normal embryonic development, feeding can be used to promote larval settlement and increase the survival rate.

## 5. Conclusions

In recent years, the harsh environment caused by the greenhouse effect and heavy rains has led to the vulnerability and death of *B. violacea*. Through the application of this research, the stress response of corals due to temperature and salinity changes can be effectively alleviated, with significant improvements in coral survival, growth, larval settlement and embryonic development. This study is a preliminary discussion, and in the future, more species in coral restoration or the application of large-scale coral aquaculture can be promoted to achieve the goal of sustainable coral development. This technology has been applied to TCK coral farm in Taiwan to prevent threats caused by changes in temperature or salinity during *B. violacea* cultivation.

## Figures and Tables

**Table 1:** The water quality conditions in the study.

Water Quality Conditions	Water Quality Data
pH	8.0 ± 0.31
Ammonia nitrogen	0.04 ± 0.06 mg/L
Nitrous acid	0.02 ± 0.04 mg/L
Nitric acid	0.10 ± 0.01 mg/L
Calcium	400 ± 44.05 ppm
Magnesium	1280 ± 75.03 ppm
Phosphate	0.02 ± 0.01 ppm

**Table 2:** Feed nutrition and changes in body composition 1 h after feeding of coral.

Nutritional Indicators	Protein	Lipid	Glucose
	(µg/mg)	(µg/mg)	(µg/mg)
Nutrient composition	76.69 ^a^	6.04 ^c^	19.00 ^b^
(Feed nutrition)	(1.49)	(0.03)	(0.32)
No feeding	305.12 ^b^	1.11 ^b^	2.41 ^c^
(Coral body composition)	(14.23)	(0.31)	(0.36)
1 h after being fed	403.13 ^a^	1.31 ^b^	3.72 ^b^
(Coral body composition)	(19.20)	(0.08)	(0.24)

Different Latin alphabet letters indicate significant differences among groups (*p* < 0.05). Values are expressed as means ± SDs (*n* = 3).

**Table 3:** The effect of feeding on *Briareum violacea* growth under temperature and salinity stress.

Salinity and Temperature													
		25 psu			30 psu			35 psu			40 psu		
Feeding		20 °C	25 °C	30 °C	20 °C	25 °C	30 °C	20 °C	25 °C	30 °C	20 °C	25 °C	30 °C
Y	Net Increase	0.00	3.67 ^c^	8.00 ^b^	4.00 ^c^	13.00 ^a^	9.00 ^b^	11.00 ^ab^	14.33 ^a^	4.00 ^c^	2.00 ^d^	1.67 ^d^	2.67 ^d^
	(Number ± 95%)	(0.00)	(0.58)	(1.00)	(1.00)	(2.00)	(1.00)	(1.00)	(1.53)	(1.00)	(1.00)	(0.58)	(0.58)
N	Net Increase	0.00	0.00	0.00	0.00	9.33 ^a^	8.33 ^a^	9.00 ^a^	9.33 ^a^	0.00	0.00	0.00	0.00
	(Number ± 95%)	(0.00)	(0.00)	(0.00)	(0.00)	(1.15)	(1.53)	(1.00)	(0.58)	(0.00)	(0.00)	(0.00)	(0.00)

Different Latin alphabet letters indicate significant differences among groups (*p* < 0.05). The values are expressed as the means ± SDs (*n* = 30 colonies). Y: feeding treatment group, N: nonfeeding treatment group.

**Table 4:** The effect of feeding on *Briareum violacea* zooxanthellae, chlorophyll *a* and survival during temperature and salinity stress.

Salinity and Temperature													
		25 psu			30 psu			35 psu			40 psu		
Feeding		20 °C	25 °C	30 °C	20 °C	25 °C	30 °C	20 °C	25 °C	30 °C	20 °C	25 °C	30 °C
Y	Zooxanthellae	0.7 ^h^	1.7 ^e^	1.0 ^g^	1.50 ^f^	4.3 ^b^	3.13 ^d^	3.72 ^c^	4.82 ^a^	1.73 ^e^	0.30 ^i^	0.15 ^i^	0.13 ^i^
	(cell × 10^7^ cm^−2^)	(0.04)	(0.03)	(0.02)	(0.05)	(0.07)	(0.03)	(0.03)	(0.02)	(0.06)	(0.05)	(0.03)	(0.05)
	Chlorophyll *a*	1.21 ^e^	2.67 ^e^	1.42 ^e^	14.04 ^d^	43.70 ^b^	41.21 ^b^	32.21 ^c^	51.23 ^a^	14.30 ^d^	1.13 ^e^	1.19 ^e^	1.17 ^e^
	(μg cm^−2^)	(0.01)	(0.25)	(0.42)	(1.22)	(2.30)	(1.42)	(1.01)	(1.03)	(1.41)	(1.02)	(0.38)	(0.53)
	Survival rate	100	100	100	100	100	100	100	100	100	100	100	100
	(%)	(0.00)	(0.00)	(0.00)	(0.00)	(0.00)	(0.00)	(0.00)	(0.00)	(0.00)	(0.00)	(0.00)	(0.00)
N	Zooxanthellae	0.07 ^d^	0.13 ^d^	0.12 ^d^	0.08 ^d^	4.21 ^a^	2.30 ^b^	1.74 ^c^	4.30 ^a^	0.13 ^d^	0.17 ^d^	0.23 ^d^	0.17 ^d^
	(cell × 10^7^ cm^−2^)	(0.01)	(0.01)	(0.02)	(0.02)	(0.05)	(0.03)	(0.01)	(0.04)	(0.02)	(0.02)	(0.02)	(0.03)
	Chlorophyll *a*	1.09 ^f^	0.96 ^f^	0.95 ^f^	0.72 ^f^	42.33 ^b^	38.21 ^c^	19.21 ^d^	49.24 ^a^	10.03 ^e^	1.02 ^f^	1.01 ^f^	1.04 ^f^
	(μg cm^−2^)	(0.02)	(0.04)	(0.02)	(0.01)	(1.21)	(1.23)	(1.73)	(2.31)	(1.20)	(0.01)	(0.01)	(0.01)
	Survival rate	46.67 ^b^	53.33 ^b^	53.33 ^b^	73.00 ^ab^	100 ^a^	100 ^a^	73.33 ^a^	100 ^a^	73.33 ^ab^	46.67 ^b^	33.33 ^bc^	26.67 ^bc^
	(%)	(11.55)	(11.55)	(11.55)	(11.55)	(0.00)	(0.00)	(11.55)	(0.00)	(11.55)	(11.55)	(11.55)	(11.55)

Different Latin alphabet letters indicate significant differences among groups (*p* < 0.05). The values are expressed as the means ± SDs (*n* = 30 colonies). Y: feeding treatment group, N: nonfeeding treatment group.

**Table 5:** The effect of feeding on *Briareum violacea* larval zooxanthellae, chlorophyll *a* and survival during temperature and salinity stress.

Salinity and Temperature													
		25 psu			30 psu			35 psu			40 psu		
Feeding		20 °C	25 °C	30 °C	20 °C	25 °C	30 °C	20 °C	25 °C	30 °C	20 °C	25 °C	30 °C
Y	Zooxanthellae	0.00	0.71 ^g^	1.02 ^d^	0.00	2.13 ^a^	0.83 ^f^	0.00	2.22 ^a^	1.43 ^b^	0.00	0.93 ^e^	0.85 ^f^
	(cell × 10^7^ cm^−2^)	(0.00)	(0.01)	(0.02)	(0.00)	(0.03)	(0.03)	(0.00)	(0.02)	(0.06)	(0.00)	(0.03)	(0.02)
	Chlorophyll *a*	0.00	0.07 ^b^	0.02 ^c^	0.00	0.11 ^a^	0.05 ^b^	0.00	0.11 ^a^	0.09 ^ab^	0.00	0.08 ^ab^	0.07 ^ab^
	(μg cm^−2^)	(0.00)	(0.01)	(0.01)	(0.00)	(0.01)	(0.03)	(0.00)	(0.02)	(0.01)	(0.00)	(0.01)	(0.03)
	Survival rate	0.00	33.33 ^b^	20.00 ^b^	0.00	36.67 ^b^	33.33 ^b^	0.00	45.00 ^a^	30.00 ^b^	0.00	33.33 ^b^	25.00 ^b^
	(%)	(0.00)	(5.77)	(10.00)	(0.00)	(5.77)	(5.77)	(0.00)	(5.00)	(10.00)	(0.00)	(5.77)	(5.00)
N	Zooxanthellae	0.00	0.53 ^b^	0.11 ^d^	0.00	0.54 ^b^	0.20 ^c^	0.00	1.20 ^a^	0.13 ^d^	0.00	0.11 ^d^	0.00
	(cell × 10^7^ cm^−2^)	(0.00)	(0.01)	(0.01)	(0.00)	(0.05)	(0.03)	(0.00)	(0.04)	(0.02)	(0.00)	(0.01)	(0.00)
	Chlorophyll *a*	0.00	0.04 ^ab^	0.05 ^ab^	0.00	0.04 ^ab^	0.04 ^ab^	0.00	0.06 ^a^	0.03 ^c^	0.00	0.05 ^ab^	0.00
	(μg cm^−2^)	(0.00)	(0.01)	(0.01)	(0.00)	(0.01)	(0.01)	(0.00)	(0.01)	(0.01)	(0.00)	(0.01)	(0.00)
	Survival rate	0.00	25.00 ^b^	8.33 ^d^	0.00	28.33 ^b^	15.00 ^c^	0.00	38.33 ^a^	40.00 ^a^	0.00	33.33 ^a^	0.00
	(%)	(0.00)	(5.00)	(2.89)	(0.00)	(2.89)	(5.00)	(0.00)	(7.64)	(5.00)	(0.00)	(5.77)	(0.00)

Different Latin alphabet letters indicate significant differences among groups (*p* < 0.05). The values are expressed as the means ± SDs (*n* = 30 larvae). Y: feeding treatment group, N: nonfeeding treatment group.

## Data Availability

Not applicable.
